# Palliative Care for Community-Dwelling Persons Living With Dementia: A Concept Analysis

**DOI:** 10.1093/geroni/igaf122.1698

**Published:** 2025-12-31

**Authors:** Xuefan Ji

**Affiliations:** Columbia University, New York, New York, United States

## Abstract

**Background:**

Palliative care is underutilized by patients with neurological conditions, especially persons living with dementia (PLWD) residing in community settings in the U.S. Palliative care has not been defined for community-dwelling PLWD, and misconceptions that palliative care is only applicable when the patient is dying may have led to underutilization in this population.

**Aim:**

To clarify and provide a refined definition of the concept of palliative care for community-dwelling PLWD.

**Method:**

A concept analysis using Rodger’s method was completed. Three databases were searched for literature that described the antecedents, defining attributes, and consequences of palliative care in the context of community-dwelling PLWD. A model case of the concept was identified from the literature.

**Results:**

A total of 16 studies were included. Palliative care differs from hospice and does not require PLWD to be near end of life. Antecedents included acute care use, poor prognosis, and primary care provider referral. Defining attributes encompassed comprehensive care, care coordination, and serious illness communication. Consequences included more home death, less hospitalization and other burdensome acute care use, and patients and caregivers electing less aggressive care option.

**Discussion:**

Antecedents of acute care use and poor prognosis may suggest late utilization of palliative care among PLWD and lack of palliative care needs screening. Defining attribute of care coordination suggest primary care providers (PCPs) play a crucial role in coordinating palliative care for PLWD. Future study should investigate the concept of palliative care needs in PLWD and effectiveness of primary care based palliative care models for PLWD

